# Nanocarbon or indocyanine green: Which is superior for gasless transaxillary endoscopic thyroidectomy to protect the parathyroid gland?

**DOI:** 10.3389/fsurg.2022.1035840

**Published:** 2022-11-10

**Authors:** Zhipeng Ye, Keren Wu, Zhao Hu, Fa Jin

**Affiliations:** First School of Clinical Medicine, Zhejiang Chinese Medical University, Hangzhou, China

**Keywords:** ICG angiography, parathyroid, gasless insufflation transaxillary endoscopic thyroidectomy, nano-carbon, thyroid cancer

## Abstract

**Background:**

Damage to the parathyroid glands remains a frequent complication after thyroidectomy, often resulting in hypoparathyroidism. Accordingly, identifying the parathyroid glands during thyroid surgical procedures is indispensable to prevent accidental surgical removal.

**Methods:**

The participants were randomly divided into three groups (indocyanine green [ICG], nanocarbon [NC], and control group). To identify and protect parathyroid glands during neck lymph node dissection in patients with thyroid cancer, IG was intravenously administered to the ICG group, whereas the NC group received an intra-thyroid injection of the NC suspension before dissection. IG was intravenously administered to each group after dissection. Subsequently, we analyzed surgical outcomes, including operative time, number of lymph nodes, serum calcium, and number of parathyroid glands.

**Results:**

We included 30 patients who underwent gasless transaxillary endoscopic thyroidectomy for thyroid cancer. Based on our findings, a greater number of parathyroid glands (*P *< 0.01) and higher postoperative parathyroid hormone (PTH) levels were detected in the NC and ICG groups than those in the control group (*P* < 0.01). The number of parathyroid glands and postoperative PTH levels in the NC group were higher than those in the ICG group (*P *< 0.01).

**Conclusions:**

Gasless transaxillary endoscopic thyroidectomy with NC and ICG for thyroid cancer could effectively protect the parathyroid gland and afford satisfactory clinical efficacy. NC could offer an advantage over ICG for protecting the parathyroid gland.

## Introduction

The number of patients undergoing thyroid surgery has steadily grown in recent years (1, 2), with the rate of new thyroid cancer cases increasing by an average of 4.5% annually (3). Iatrogenic injury of the parathyroid gland post-thyroidectomy remains a common complaint. Damage to the parathyroid glands often leads to hypoparathyroidism, accounting for up to 11% of cases and necessitating calcium and vitamin D supplementation (4). Among patients who underwent permanent hypoparathyroidism, the mortality rate is 2.2%, which is significantly higher than that in patients without permanent hypoparathyroidism (5). Identifying the parathyroid glands during thyroid-related surgical procedures remains crucial to prevent accidental surgical removal and iatrogenic devascularization.

Several intraoperative procedural techniques, such as indocyanine green (ICG) fluorescence and nanocarbon (NC), have been explored to distinguish the parathyroid gland and were found to be safe and feasible (6, 7). Carbon nanoparticles or NCs exhibit a characteristically high degree of lymphatic system tropism, tracing speed, rate of black staining, and high color contrast with the surrounding tissue (8) and can be used to identify the parathyroid during thyroid surgery (9). Angiography using ICG has been used to identify sentinel lymph nodes and parathyroid glands, as well as to evaluate the vascular blood flow of the parathyroid glands (10, 11).

Transaxillary endoscopic thyroidectomy (ET) affords a concealed incision and clear vision and has emerged as an alternative to conventional open thyroidectomy, with superior cosmetic results (12–14). Previous studies assessing ET combined with ICG or NC have compared surgical and oncologic outcomes, reporting similar or superior outcomes to those without ICG or NC (6, 7). However, few studies have compared parathyroid gland protection and hypoparathyroidism or hypocalcemia between transaxillary ET using ICG or NC.

Therefore, we aimed to compare the feasibility of near-infrared fluorescence (NIRF) imaging with ICG and contrast development with NC to identify the parathyroid gland and assess its correlation with parathyroid gland NIRF imaging with ICG and postoperative complications, including hypoparathyroidism or hypocalcemia.

## Patients and methods

### Patients

This study was conducted at the Department of Surgery, the First Affiliated Hospital of Zhejiang Chinese Medicine University (ZCMU), between December 2021 and December 2022.

The study protocol was approved by the ethics committee of the First Affiliated Hospital of ZCMU. The reference number for study approval was 2021-KL-046-01. The protocol was registered at Chictr.org.cn (registration number ChiCTR2100049512).

**Figure 1:**
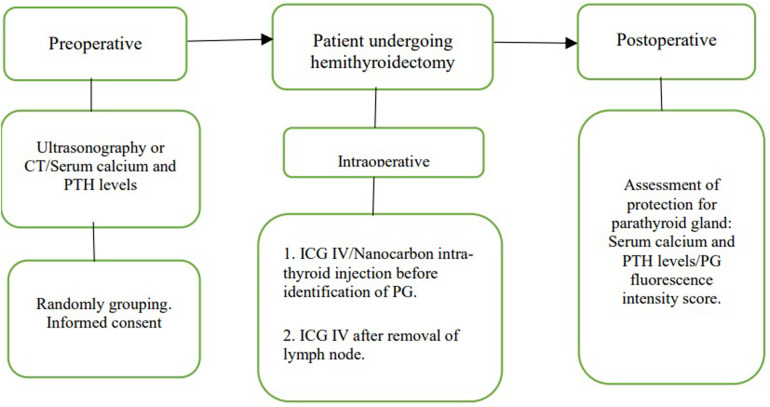
Flowchart of the study procedures.

Inclusion criteria for study participation were as follows: consecutive male and female patients, aged ≥18 years, scheduled for hemithyroidectomy and central cervical lymph node dissection, with normal liver and renal function, and who provided written informed consent. Selected participants with a pathological diagnosis of papillary thyroid carcinoma (tumor size <1.0 cm) were randomly divided into three groups. Preoperative ultrasonography and computed tomography revealed no lateral lymph node metastases or papillary thyroid carcinoma. The flowchart of the study procedure is shown in Figure 1.

### Operative methods

All thyroid surgeries were performed by the same surgeon using a modified operative technique and under general anesthesia. The standard operation was performed as reported by Kwak et al. (15). The recurrent laryngeal nerve (RLN) was systematically examined and identified by intraoperative neuromonitoring.

To identify and protect the parathyroid glands during neck lymph node dissection in patients with thyroid cancer, ICG angiography (7.5 mg/3 ml, intravenously) was performed intraoperatively (ICG group), whereas the NC suspension (0.2 ml) was injected into the thyroid gland over 2 min (NC group). After neck lymph node dissection, each group underwent ICG angiography to assess the vascularization of the parathyroid gland. ICG vascularization was scored using a previously established methodology: 0 (no vascularization), 1 (moderate vascularization), and 2 (excellent vascularization).

Intraoperative changes in serum parathyroid hormone (PTH) levels were measured at the start and end of the surgery, and blood loss, length of stay, vocal cord injury, and clinical manifestation of hypocalcemia were also examined to assess postoperative outcomes. Additional data were collected to establish a database and record the surgical approach, patient sex and age, largest tumor diameter, operative time, postoperative drainage volume, the total number of central lymph nodes, and the number of metastatic central lymph nodes.

### Statistical analysis

All statistical calculations were conducted using SPSS (version 22.0; IBM Corp., Armonk, NY, USA). Statistical analyses were performed using the chi-square test and analysis of variance test, where appropriate, with significance set at *P* < 0.05. Measurement data are expressed as the sample mean ± standard deviation $\left(\bar{\chi }\pm s\right)$.

## Results

From January 2022 to June 2022, 30 patients who underwent transaxillary ET were selected randomly for inclusion in the present study. Table 1 summarizes the demographic characteristics of all patients included in the analysis. We detected no significant differences in average age (*P *= 0.405), sex (*P *= 0.787), body mass index (*P *= 0.833), tumor size (*P *= 0.476), and tumor location (*P *= 0.873).

**Table 1:** Demographic and clinical data of included patients.

	Control (*n* = 10)	NC (*n* = 10)	ICG (*n* = 10)	*P*-value
**Gender**				
Female/male	8/2	8/2	9/1	0.787
Age (years)	37.50 ± 7.028	36.50 ± 7.81	33.30 ± 6.634	0.405
BMI (kg/m^2^)	22.28 ± 2.03	22.08 ± 3.67	21.53 ± 2.61	0.833
Tumor size, mm	6.40 ± 1.96	7.20 ± 31.68	6.30 ± 1.70	0.476
**Tumor location**				
Left/right	5/5	4/6	4/6	0.873

BMI, body mass index; ICG, indocyanine green; NC, nanocarbon.

Operative time, blood loss, drainage content, and hospitalization days did not significantly differ between groups (*P *= 0.826, *P *= 0.805, *P *= 0.797, and *P *= 0.160, respectively). Five cases of transient hoarseness were documented in all groups, which resolved within 3 months. Permanent RLN palsy was not observed in any patient. One patient in the control group, who underwent right hemithyroidectomy with central neck dissection, experienced acroagnosis. The overall complication rate did not significantly differ between groups (*P *= 0.535). There were no significant differences in the number of lymph node metastases and the total number of lymph node dissections among examined groups (*P *= 0.354 and *P *= 0.305) (Table 2).

**Table 2:** Perioperative data regarding gasless insufflation transaxillary endoscopic thyroidectomy.

	Control (*n* = 10)	NC (*n* = 10)	ICG (*n* = 10)	*P-*value
Operative time, min	146.70 ± 30.68	22.08 ± 28.53	154.0 ± 17.79	0.826
Intraoperative blood loss	10.50 ± 5.50	12.50 ± 8.25	11.50 ± 6.26	0.805
Hospital stay, days	6.40 ± 1.08	5.40 ± 0.70	6.00 ± 1.49	0.160
Postoperative drainage, ml	211.0 ± 35.10	203.0 ± 39.03	198.5 ± 50.00	0.797
Harvested LNs, *n*	5.60 ± 1.58	7.10 ± 2.92	5.40 ± 3.13	0.305
Metastatic LNs, *n*	0.70 ± 1.25	1.70 ± 2.21	0.80 ± 1.40	0.354
Postoperative complications, Nonexis-tence/existence	7/3	9/1	8/2	0.535

ICG, indocyanine green; LNs, lymph nodes; NC, nanocarbon.

**Figure 2:**
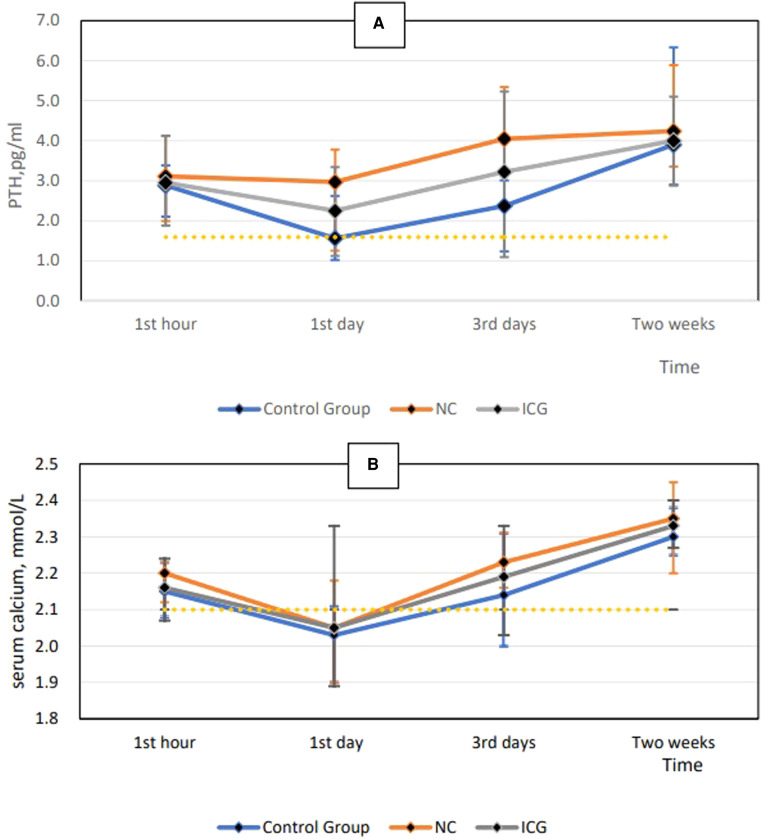
Postoperative PTH level at each time point (**A**) and postoperative serum calcium level at each time point (**B**). PTH, parathyroid hormone.

As shown in Table 3, parathyroid glands were present in all patients. The NC and ICG groups displayed a higher number of parathyroid glands and total ICG scores, with a lower incidence of accidental parathyroid excision and temporary hypoparathyroidism than the control group (*P *< 0.05). The number of parathyroid glands and the total ICG score were higher in the NC group than those in the ICG group (*P *< 0.05). Permanent hypoparathyroidism did not occur in any group. There were no significant differences in preoperative serum calcium and PTH levels across examined groups. In the NC group, postoperative PTH and serum calcium levels in all patients returned to normal levels within three days (*P *= 0.923 and *P *= 0.931, respectively); in the control group, normal levels were restored within two weeks (Figures 2A,B).

**Table 3:** Perioperative period data of parathyroid function.

		All participants (*n* = 30)	Control (*n* = 10)	NC (*n* = 10)	ICG (*n* = 10)	Statistical values	*P* value
Number of parathyroid glands, *n*		1.50 ± 0.509	1.10 ± 0.316	1.90 ± 0.316	1.50 ± 0.527	*F* = 10.047	0.001
Preoperative serum c-alcium, mmol/l		2.31 ± 0.09	2.30 ± 0.10	2.32 ± 0.09	2.32 ± 0.10	*F* = 0.081	0.923
Serum calcium postoperatively, mmol/L	1st hour	2.16 ± 0.06	2.15 ± 0.06	2.20 ± 0.04	2.16 ± 0.07	*F* = 1.570	0.226
	1st day	2.05 ± 0.09	2.03 ± 0.06	2.05 ± 0.10	2.05 ± 0.10	*F* = 0.097	0.908
	3rd days	2.18 ± 0.10	2.14 ± 0.10	2.23 ± 0.05	2.19 ± 0.10	*F* = 2.426	0.107
	Two weeks	2.33 ± 0.07	2.30 ± 0.06	2.35 ± 0.05	2.33 ± 0.08	*F* = 1.035	0.369
Preoperative PTH, pg/ml		4.46 ± 1.16	4.37 ± 1.53	4.42 ± 0.82	4.57 ± 1.13	*F* = 0.072	0.931
Postoperative PTH, pg/ml	1st hour	2.98 ± 0.68	2.88 ± 0.67	3.11 ± 0.63	2.95 ± 0.78	*F* = 0.290	0.750
	1st day	2.26 ± 0.92	1.56 ± 0.46	2.97 ± 0.92	2.25 ± 0.76	*F* = 9.098	0.001
	3rd days	3.21 ± 1.12	2.37 ± 0.69	4.05 ± 0.73	3.22 ± 1.20	*F* = 8.754	0.001
	Two weeks	4.07 ± 0.87	3.90 ± 1.19	4.24 ± 0.69	4.00 ± 0.68	*F* = 0.526	0.797
Total ICG score		3.0 ± 1.129	2.0 ± 0.667	3.9 ± 0.994	3.0 ± 0.816	*F* = 12.905	0.000

Total score, Sum total of parathyroid glands ICG scores.

ICG, indocyanine green; NC, nanocarbon; PTH, parathyroid hormone.

As shown in Table 4, the postoperative biochemical profile, including levels of serum calcium and PTH alterations, was measured at 1 h postoperatively in all patients. No significant differences in PTH alterations or symptomatic postoperative hypocalcemia were detected for different ICG vascularization scores. Furthermore, we noted no significant difference in serum calcium levels.

**Table 4:** ICG vascularization score and postoperative parathyroid function.

ICG score	<2	2–4	≥4	*P*-value
Serum calcium, mmol/L	2.09 ± 0.00	2.05 ± 0.09	2.03 ± 0.09	0.649
PTH, pg/ml (range)	1.51 ± 0.23	2.11 ± 0.87	2.72 ± 0.99	0.132

ICG, indocyanine green; PTH, parathyroid hormone.

## Discussion

Postoperative hypoparathyroidism is the most common complication associated with total thyroidectomy (16). In previous reports assessing thyroid surgery, the incidence of temporary hypoparathyroidism ranged between 14% and 60% (17, 18). Accordingly, developing a reliable tool to protect the parathyroid gland and prevent hypocalcemia remains crucial. Staining with NC or ICG angiography could reduce the possibility of accidental parathyroid excision, thereby decreasing the occurrence of hypoparathyroidism, while facilitating the rapid restoration of PTH and calcium levels (19, 20). However, which of these two modalities can be deemed superior for protecting the parathyroid gland?

Herein, we detected no significant differences between groups in terms of intraoperative and postoperative blood loss, operative time, hospital stay, postoperative complications, and other examined parameters. Therefore, gasless insufflation transaxillary ET combined could be feasibly and safely performed with NC and ICG imaging.

**Figure 3:**
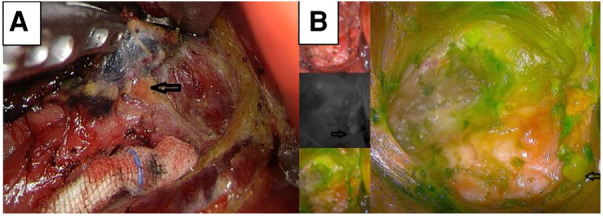
NC-stained black lymph nodes and high color contrast with the surrounding tissue for locating parathyroid (**A**). Green-stained parathyroid located by ICG (**B**). ICG, indocyanine green; NC, nanocarbon.

Establishing the anatomical position and vascular supply of parathyroid glands is essential to avoid hypoparathyroidism after thyroid surgery (21). During lymph node dissection, intravenous ICG can aid in identifying the parathyroid gland through immediate parathyroid gland angiography and green staining of the parathyroid gland. Removing the unstained adipose lymphatic tissue can protect the parathyroid gland from damage and prevent the occurrence of postoperative hypocalcemia (Figure 3B). However, ectopic parathyroids embedded in adipose tissue or thyroid tissue cannot be stained green or identified by ICG; these are often removed or damaged accidentally, leading to hypocalcemia after thyroidectomy.

NCs exhibit a markedly high degree of lymphatic system tropism, tracing speed, rate of black staining, and high color contrast with the surrounding tissue (8). When NCs were used in thyroid surgery, black-stained lymph nodes were dissected. We detected no significant differences in the number of lymph nodes or metastatic lymph nodes between the two groups. Reportedly, the dissection of sentinel lymph nodes with NCs is effective and safe for thyroid cancer surgery (22). Black-stained lymph nodes can indicate the position of adipose tissue and parathyroid glands and prevent the removal of parathyroid glands within the remaining tissue damage (Figure 3A). Likewise, it has been reported that NCs can be used to identify the parathyroid gland during thyroid surgery (9, 19). Careful identification and removal of black-stained lymphatic tissues during thyroidectomy with neck lymph node dissection can ensure complete lymph node dissection and prevent parathyroid damage, thereby effectively reducing the incidence of hypoparathyroidism. Consequently, the findings of the present study suggest that staining with NCs combined with gasless insufflation transaxillary ET can better reduce the possibility of accidental parathyroid gland excision.

In addition to a good safety profile, intravenous ICG is rapidly excreted into the bile. The short half-life of ICG in systemic circulation allows repeated application for identifying the parathyroid gland during thyroid surgery. It has been suggested that NC extravasation can impact the surgical field and the surgery. Herein, we found that the integrity of the thyroid capsule during surgery can prevent the staining of other tissues. Even if NCs spilled over and were removed gradually, the surgical field under the endoscope remained clear.

Accumulated evidence suggests that ICG angiography enables early, direct evaluation of the parathyroid glands and could help the surgeon decide whether to autotransplant a devascularized parathyroid gland. Calcium and/or PTH measurements may no longer be necessary for patients with at least one well-perfused parathyroid gland, as demonstrated using ICG angiography after thyroidectomy (20). However, we found no direct relationship between intraoperative parathyroid imaging grade and postoperative hypocalcemia or decreased PTH levels. Conversely, hypocalcemia was detected at every parathyroid imaging grade. However, the blood supply to the contralateral parathyroid glands remains intact during unilateral thyroid surgery, and hypocalcemia can recur. Calcium and/or PTH measurements are necessary for patients and cannot be replaced by ICG angiography. As a method for evaluating parathyroid gland autotransplantation, ICG remains debatable. Interestingly, high parathyroid imaging scores were found to return to normal shortly after surgery.

This study has certain limitations. First, the sample size of the present study was small, and the results need to be validated in a larger patient population. Second, randomized controlled trials assessing parathyroid autotransplantation should be conducted to clarify the relationship between ICG angiography and autotransplantation. Finally, given that unilateral thyroid surgery was performed and the contralateral parathyroid gland was intact, the accuracy of the present study may be uncertain.

## Conclusions

In summary, gasless transaxillary ET with NC and ICG for thyroid cancer can protect the parathyroid gland and provide satisfactory clinical effects. NC was superior to ICG in protecting the parathyroid gland. Intraoperative ICG angiography can be used to assess postoperative parathyroid function and serum calcium recovery; however, it is not recommended as a standard for parathyroid autotransplantation or a substitute for postoperative serum calcium/PTH measurements.

## Data Availability

The datasets presented in this study can be found in online repositories. The names of the repository/repositories and accession number(s) can be found in the article/Supplementary Material.
